# Enhancing Bone Formation Through bFGF-Loaded Mesenchymal Stromal Cell Spheroids During Fracture Healing in Mice

**DOI:** 10.3390/bioengineering11101041

**Published:** 2024-10-18

**Authors:** Kugo Takeda, Hiroki Saito, Shintaro Shoji, Hiroyuki Sekiguchi, Mitsuyoshi Matsumoto, Masanobu Ujihira, Masayuki Miyagi, Gen Inoue, Masashi Takaso, Kentaro Uchida

**Affiliations:** 1 Department of Medical Engineering and Technology, Graduate School of Medical Sciences, Kitasato University, 1-15-1 Minami-ku, Kitasato, Sagamihara 252-0375, Kanagawa, Japan; takeda.kugo@st.kitasato-u.ac.jp (K.T.); uj@kitasato-u.ac.jp (M.U.); 2 Department of Orthopaedic Surgery, Kitasato University School of Medicine, 1-15-1 Minami-ku, Kitasato, Sagamihara 252-0374, Kanagawa, Japan; kgka.condition-green@xd6.so-net.ne.jp (H.S.); catch_the_rainbow_914@yahoo.co.jp (S.S.); kwangyu3244@gmail.com (M.M.); masayuki008@aol.com (M.M.); ginoue@kitasato-u.ac.jp (G.I.); mtakaso@kitasato-u.ac.jp (M.T.); 3 Research Institute, Shonan University of Medical Sciences, Nishikubo 500, Chigasaki 253-0083, Kanagawa, Japan; guccyon_s@yahoo.co.jp

**Keywords:** mesenchymal stromal cells, spheroid, basic fibroblast growth factor, fracture healing

## Abstract

This study aimed to evaluate the osteogenic potential of mesenchymal stromal cell (MSC) spheroids combined with the basic fibroblast growth factor (bFGF) in a mouse femur fracture model. To begin, MSC spheroids were generated, and the expression of key trophic factors (*bFGF Bmp2*, and *Vegfa*) was assessed using quantitative PCR (qPCR). A binding assay confirmed the interaction between the bFGF and the spheroids’ extracellular matrix. The spheroid cultures significantly upregulated *bFGF*, *Bmp2*, and *Vegfa* expression compared to the monolayers (*p* < 0.001), and the binding assay demonstrated effective bFGF binding to the MSC spheroids. Following these in vitro assessments, the mice were divided into five groups for the in vivo study: (1) no treatment (control), (2) spheroids alone, (3) bFGF alone, (4) bFGF-loaded spheroids (bFGF-spheroids), and (5) non-viable (frozen) bFGF-loaded spheroids (bFGF-dSpheroids). Bone formation was analyzed by a micro-CT, measuring the bone volume (BV) and bone mineral content (BMC) of the mice four weeks post-fracture. A high dose of the bFGF (10 µg) significantly promoted bone formation regardless of the presence of spheroids, as evidenced by the increases in BV (bFGF, *p* = 0.010; bFGF-spheroids, *p* = 0.006; bFGF-dSpheroids, *p* = 0.032) and BMC (bFGF, *p* = 0.023; bFGF-spheroids, *p* = 0.004; bFGF-dSpheroids, *p* = 0.014), compared to the controls. In contrast, a low dose of the bFGF (1 µg) combined with the MSC spheroids significantly increased BV and BMC compared to the control (BV, *p* = 0.012; BMC, *p* = 0.015), bFGF alone (BV, *p* = 0.012; BMC, *p* = 0.008), and spheroid (BV, *p* < 0.001; BMC, *p* < 0.001) groups. A low dose of the bFGF alone did not significantly promote bone formation (*p* > 0.05). The non-viable (frozen) spheroids loaded with a low dose of the bFGF resulted in a higher BV and BMC compared to the spheroids alone (BV, *p* = 0.003; BMC, *p* = 0.017), though the effect was less pronounced than in the viable spheroids. These findings demonstrate the synergistic effect of the bFGF and MSC spheroids on bone regeneration. The increased expression of the BMP-2 and VEGF observed in the initial experiments, coupled with the enhanced bone formation in vivo, highlight the therapeutic potential of this combination. Future studies will aim to elucidate the underlying molecular mechanisms and assess the long-term outcomes for bone repair strategies.

## 1. Introduction

Bone regeneration remains a significant medical challenge, as inadequate healing can lead to complications such as trauma, tumor formation, or infections like osteomyelitis. Mesenchymal stem/stromal cells (MSCs), known for their ability to differentiate into various cell types, including osteoblasts and chondrocytes, have emerged as a promising option for bone repair [1]. In addition to their differentiation potential, MSCs secrete trophic factors essential for bone regeneration, making them a valuable resource in the development of bone repair strategies [2,3].

However, the traditional use of two-dimensional (2D) cell cultures often fails to replicate the complex in vivo conditions, leading to a reduction in crucial cellular properties such as self-renewal and differentiation potential. In contrast, three-dimensional (3D) spheroid cultures provide a more physiologically relevant environment, preserving these cellular characteristics and enhancing the secretion of trophic factors [4,5]. This makes MSC spheroids a promising tool for bone regenerative therapies [6,7,8]. Nonetheless, some studies have noted that spheroids alone may have a limited effectiveness in promoting robust bone regeneration [9,10].

A promising strategy to enhance the osteogenic potential of MSC spheroids is their combination with growth factors. Among the fibroblast growth factor (FGF) family, the basic fibroblast growth factor (bFGF) is particularly notable due to its significant accumulation in the bone matrix and its expression in the periosteum during the early stages of bone formation [11]. Studies in animal models have demonstrated that a locally applied recombinant human bFGF (rhbFGF) enhances osteogenesis in bone fractures, defects, and osteoporotic bone regeneration [12,13,14,15,16,17]. Clinical trials have further shown that bFGF accelerates bone healing following osteotomy and in tibial shaft fractures [18,19]. Despite its potential, bFGF’s efficacy diminishes rapidly due to its diffusion into body fluids, and high doses of bFGF may cause adverse effects [20,21].

To address these challenges, combining a bFGF with a suitable carrier, such as collagen, may provide a controlled release system [22,23]. The extracellular matrix including collagen, which is abundantly produced by MSC spheroids [24], has a high affinity for bFGF and can act as a natural carrier, allowing for the sustained delivery of the growth factors. This combination of bFGF and the ECM produced by MSC spheroids holds the potential to significantly improve bone regeneration by promoting localized and sustained bFGF activity.

In this study, we aim to investigate the effects of combining bFGF with MSC spheroids on bone formation during fracture healing using a mouse fracture model. By exploring the synergistic potential of growth factors and 3D spheroids, we hope to identify an effective strategy for enhancing bone regeneration.

## 2. Materials and Methods

### 2.1. Cell Culture

The mesenchymal stromal cell (MSC) line KUM10 [25,26,27], derived from the bone marrow of C57/BL6 mice to mimic autologous transplantation, was obtained from the RIKEN BioResource Center (Tsukuba, Ibaraki, Japan). The expression of mesenchymal stem cell markers in KUM10 was analyzed using flow cytometry, as described below. The KUM10 cells were cultured in minimum essential medium alpha, supplemented with 10% fetal bovine serum (FBS) and 1% penicillin/streptomycin (Sigma-Aldrich, St. Louis, MO, USA), and incubated at 37 °C in a humidified atmosphere containing 5% CO_2_. The subconfluent KUM10 cells were detached using trypsin, washed, and resuspended in a fresh culture medium. The cells were seeded into ultra-low attachment Nunclon Sphera plates (Thermo Scientific, Roskilde, Denmark) for a spheroid culture, or into conventional 96-well plates (Corning Costar, Cambridge, MA, USA) for a monolayer culture, at a density of 1.25 × 10^5^ cells per well. The cultures were maintained for 5 days to allow spheroid formation. To evaluate the consistency of spheroid formations, the spheroid size was measured across the three independent experiments. No significant differences in spheroid size were observed among the experiments, indicating uniformity in spheroid formation (Supplementary Figure S1). These spheroids were subsequently used for a quantitative PCR (qPCR) analysis, a binding assay, or fracture model experiments.

### 2.2. Flow Cytometric Analysis

To evaluate the cell surface markers for mesenchymal stem cells in the KUM10 culture, the cells were incubated with fluorescein isothiocyanate (FITC)-conjugated anti-CD45 (pan leukocyte marker), phycoerythrin (PE)-conjugated anti-CD11b (macrophage marker), anti-CD31 (endothelial cell marker), PE-conjugated anti-Sca-1 (MSC marker) [28], PE-conjugated anti-PDGFRα (MSC marker) [28], or PE-conjugated anti-CD29 (MSC marker) [29] antibodies. All antibodies were purchased from BioLegend (Sandiego, CA, USA). Flow cytometry was performed using the FACSVerse™ system (BD Biosciences, San Jose, CA, USA), recording 10,000 events per sample. The non-staining controls were used to confirm the presence of positive cells.

### 2.3. qPCR Analysis

Quantitative PCR (qPCR) was performed to compare the gene expression profiles of the monolayer and spheroid-cultured cells. Total RNA was extracted from three independent monolayer and spheroid samples, each consisting of 1.25 × 10^5^ cells cultured for 5 days, using Trizol (Thermo Scientific) in combination with the Direct-zol MicroPrep kit (Zymo Research, Orange, CA, USA). RNA concentration was measured with a spectrophotometer (Denovix, DE, USA) [4]. First-strand cDNA was synthesized using 200 ng of total RNA with Superscript III RT™ (Invitrogen, Carlsbad, CA, USA). Real-time PCR was then performed using SYBR™ Green (Qiagen, Valencia, CA, USA). The primer sequences are listed in Table 1. We selected the *Bmp2* and *Vegfa* genes due to their reported synergistic effects with the bFGF in promoting osteogenesis and angiogenesis [24,30,31]. The housekeeping gene *Gapdh* was used as an internal control, and the delta–delta Ct method was employed to calculate relative gene expression. The results were normalized to the mean expression levels in the monolayer cultures for a comparative analysis.

### 2.4. Binding Assay

The human recombinant bFGF was purchased from Kaken Pharmaceutical, Tokyo, Japan. To evaluate the binding of the bFGF to spheroids, the following protocol was applied. First, the bFGF was labeled with fluorescein isothiocyanate (FITC) using an FITC conjugation kit (Lightning-Link, Abcam, Cambridge, UK), following the manufacturer’s instructions. Eight spheroids were placed in 1.5 mL centrifuge tubes and incubated with either 0 μg (PBS control) or 1 μg of bFGF in 10 μL of PBS at 37 °C for 30 min. After incubation, the spheroids were washed twice with PBS. Fluorescence microscopy was then used to visualize the binding of the FITC-labeled bFGF to the spheroids.

To further estimate the binding capacity of the spheroids with bFGF, eight spheroids or frozen spheroids (non-viable cells) were placed in 1.5 mL centrifuge tubes and incubated with 1 μg of bFGF in 10 μL of PBS at 37 °C for 30 min. This approach facilitated the interaction between the bFGF and both the viable and non-viable spheroids, allowing for the assessment of the bFGF binding efficiency under controlled conditions. After incubation, the mixture was centrifuged at 15,000× *g* for 5 min to separate the unbound bFGF from the spheroids. The supernatant was collected, and the concentration of unbound bFGF was measured using a commercial bFGF ELISA kit (BioLegend, San Diego, CA, USA). This assay quantified the binding of the bFGF to the spheroids by determining the decrease in bFGF concentration in the supernatant after incubation.

### 2.5. Mouse Fracture Model

A femoral fracture model was established in the 10-week-old C57BL/6J mice [32]. The mice were housed at Nippon Charles River Laboratories (Kanagawa, Japan), in a semi-barrier system with controlled temperature (23 ± 2 °C), humidity (55 ± 10%), and a 12-hour light/dark cycle. They were provided standard rodent chow (CRF-1; Oriental Yeast, Tokyo, Japan). To create the fracture model, a 10 mm incision was made on the lateral side of the left thigh under sterile conditions. A lateral parapatellar incision (4 mm) was made to medially dislocate the left patella. Afterward, a 0.5 mm hole was drilled into the intercondylar notch, and a 0.5 mm diameter stainless steel needle was retrogradely inserted into the intramedullary canal for stabilization. Osteotomy was performed using a 0.22 mm wire saw via a small lateral approach. Two sets of experiments were conducted to evaluate the efficacy of the bFGF and MSC spheroids in promoting bone regeneration. In the first experiment, the animals were divided into five groups: no treatment (control), spheroids alone, 10 μg of bFGF alone, 10 μg of bFGF-loaded spheroids, and 10 μg of bFGF-loaded non-viable spheroids (dSpheroids). In the second experiment, the animals were similarly divided into five groups: no treatment (control), spheroids alone, 1 μg of bFGF alone, 1 μg of bFGF-loaded spheroids, and 1 μg of bFGF-loaded non-viable (frozen) spheroids (dSpheroids).

In both experiments, the treatment (spheroids, bFGF alone, or bFGF-loaded spheroids) was administered only once, immediately after the fracture, with no further dosing or treatment. The treatments were delivered directly to the fracture site using a micropipette to ensure the precise delivery of the spheroids or bFGF. The no-treatment groups served as baselines for normal fracture healing without any intervention, allowing for an accurate assessment of treatment efficacy in the other experimental groups.

The mice were monitored for four weeks post-treatment to assess bone regeneration. Painkillers, including NSAIDs, were not used as they are known to interfere with bone healing in animal models [33,34]. This decision was approved by our ethical committee to ensure the integrity of the bone healing process during the study.

The sample size for this study was calculated using G*Power 3.1 software (Heinrich-Heine-Universität Düsseldorf, Düsseldorf, Germany) based on the preliminary experiments with three animals per group (*n* = 3). The mean and standard deviation of the bone mineral content (BMC) between the control group and the bFGF-spheroid group were used to calculate the effect size. The effect size (Cohen’s d) was calculated to quantify the magnitude of the difference between two groups in terms of their BMC. Cohen’s d was determined by taking the difference in the mean values of the two groups and dividing it by the pooled standard deviation, which was calculated by averaging the squared standard deviations of both groups. For the high-dose (10 μg bFGF) experiment, the mean BV values for the control group were 14.75 ± 2.73, and for the bFGF-spheroid group, they were 22.30 ± 3.58. For the low-dose (1 μg bFGF) experiment, the mean BV values for the control group were 15.53 ± 4.87, and for the bFGF-spheroid group, they were 25.09 ± 8.63. Based on these effect size calculations (Cohen’s d = 2.37 for the high-dose experiment and d = 1.37 for the low-dose experiment), a significance level (α) of 0.05 and a power (1 − β) of 0.8 were used to determine the required sample sizes.

The results indicated that a sample size of 5 per group was necessary for the high-dose bFGF experiment, and 10 per group was required for the low-dose bFGF experiment. All animal experiments were conducted in accordance with the guidelines of the Animal Ethics Committee of Kitasato University (Approval number: 2023-137), ensuring the ethical treatment and proper care of the animals.

### 2.6. Determination of New Bone Volume and Bone Mineral Content

Four weeks post-treatment all mice were sacrificed, and the femurs along with the surrounding muscle tissue were harvested and fixed in 4% paraformaldehyde for 48 h at 4 °C. The femurs were then transferred to PBS and scanned using a micro-focus X-ray CT system (inspeXio SMX-90CT; Shimadzu, Tokyo, Japan) with a 90 kV acceleration voltage, 110 mA current, a voxel size of 20 μm/pixel, and a 1024 × 1024 matrix resolution. The newly developed bone volume (BV) and BMC were quantified using 3D image analysis software (Tri-3D-Bon; Ratoc System Engineering, Tokyo, Japan) on micro-CT images, focusing on a 12 mm region of interest centered at the fracture site [35,36].

### 2.7. Statistical Analysis

For the statistical analysis, the Shapiro–Wilk test was first used to assess the normality of the data. For comparisons between two groups, either the *t*-test or Mann–Whitney U test was applied depending on whether the data followed a normal distribution. For comparisons among more than two groups, the Kruskal–Wallis test with the Dunn test was used, as the data were non-parametric.

## 3. Results

### 3.1. Effect of Spheroid Size on Trophic Factor Expression

**Figure 1:**
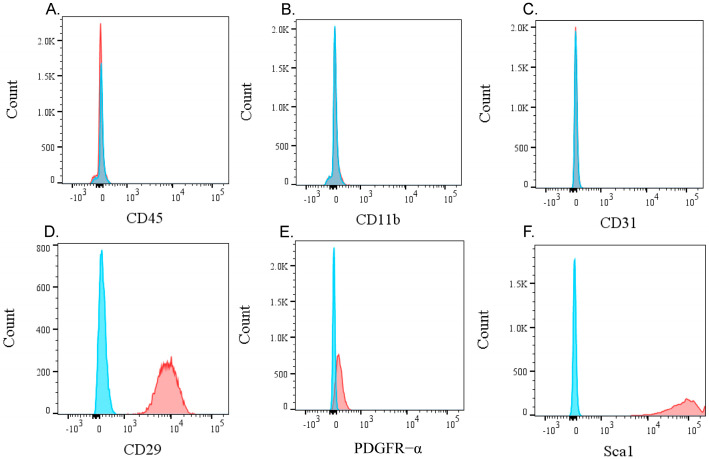
Flow cytometric analysis of KUM10 cells. Flow cytometry histograms showing the expression of surface markers in KUM10 cells. The (**A**) CD45, a pan-leukocyte marker, (**B**) CD11b, a macrophage marker, and (**C**) CD31, an endothelial cell marker, all show negative expression in KUM10 cells. In contrast, the mesenchymal stem cell markers (**D**) Sca1, (**E**) PDGFR-α, and (**F**) CD29 show positive expression in KUM10 cells. Blue histograms represent the isotype control, while red histograms represent specific antibody staining.

The KUM10 cells were negative for hematopoietic and monocytic cell markers (CD45, CD11b) and the endothelial cell marker CD31 (Figure 1A–C). In contrast, they were positive for the mesenchymal stem cell markers CD29, Sca1, and PDGFR-α (Figure 1D–F).

**Figure 2:**
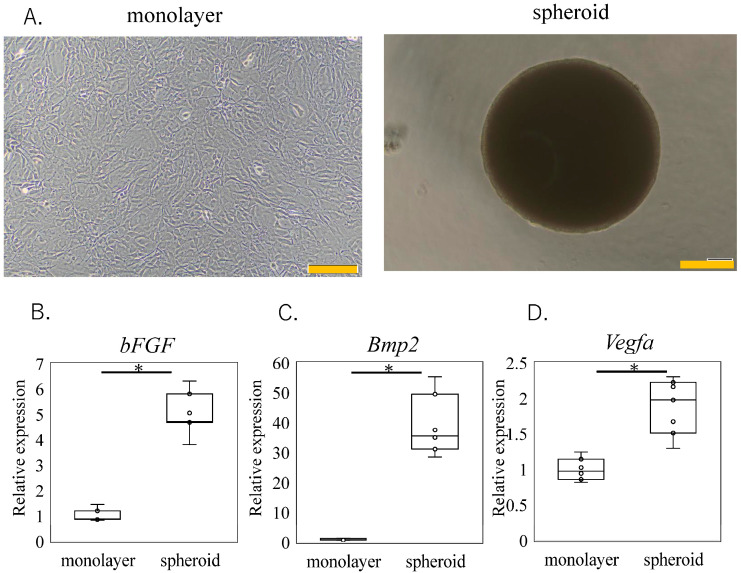
Trophic factor-related gene expression in monolayer- and spheroid-cultured KUM10 cells. (**A**) Phase-contrast microscopy images of monolayer cells and spheroids. Scale bar indicates 200 μm. (**B**–**D**) qPCR analysis results for *bFGF* (**B**), *Bmp2* (**C**), and *Vegfa* (**D**). * indicates *p* < 0.05.

The impact of a spheroid culture on the expression of the trophic factor-related genes is illustrated in Figure 2. In comparison to the monolayer cultures, the gene expressions of the *bFGF*, *Bmp2,* and *Vegfa* were significantly higher in the spheroid cultures (*Bmp2*, *p* < 0.001. *bFGF p* < 0.001 and *Vegfa*, *p* < 0.001). These findings suggest that a spheroid culture enhances the expression of certain trophic factors, such as the bFGF, BMP-2, and VEGF, which are important for bone regeneration.

### 3.2. bFGF Binding Assay

**Figure 3:**
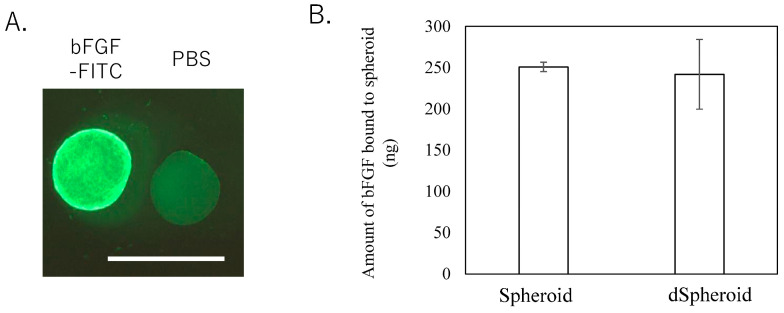
Binding assay showing (**A**) fluorescence microscopy images: scale bar indicates 1 mm. (**B**) Amount of bFGF bound to the spheroids.

Fluorescence microscopy revealed FITC fluorescence around the spheroids, confirming that the FITC-labeled bFGF successfully bound to the spheroids (Figure 3A). As demonstrated in Figure 3B, the concentration of bFGF measured in the supernatant indicated that 250.8 ± 5.7 ng and 241.9 ± 42.3 ng of the bFGF were absorbed into the spheroids and dSpheroids, respectively. There was no significant difference in the binding capacity between the two types of spheroids (Figure 3B, *p* = 0.781). These findings suggest that the bFGF effectively interacts with both viable and non-viable spheroids, highlighting their potential as growth factor carriers in bone regeneration applications.

### 3.3. Effect of High-Dose bFGF-Loaded Spheroids on Bone Volume and Bone Mineral Content After Fracture

**Figure 4:**
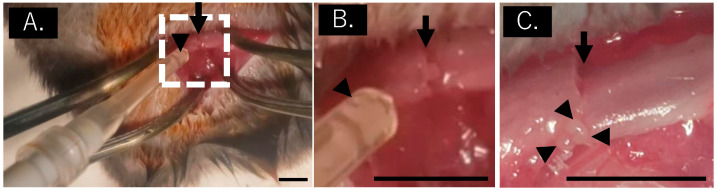
Spheroid injection into the fracture site. (**A**) Spheroid injection into the fracture site using a micropipette. The arrow indicates the fracture line, and the arrowheads point to the injected spheroids. (**B**) Enlarged view of the area highlighted by the white dashed box in (**A**). The arrowhead indicates the spheroids. (**C**) Post-injection image of the fracture site. The arrow indicates the fracture line, and the arrowheads point to the injected spheroids. Scale bar indicates 5 mm.

**Figure 5:**
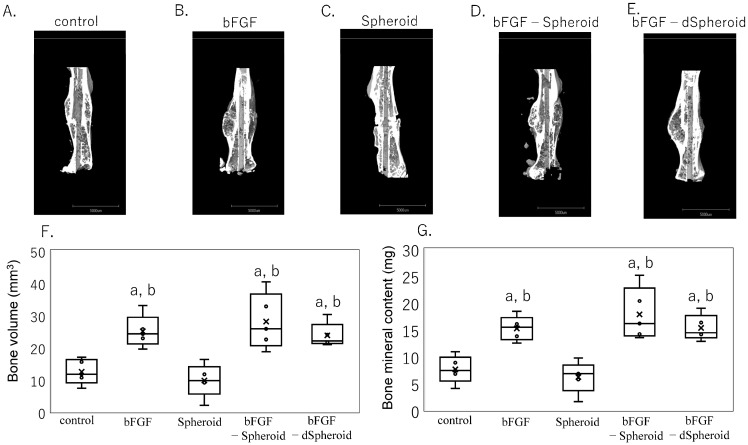
Micro-CT analysis of femurs following transplantation of high-dose bFGF-loaded spheroids. Representative 3D micro-CT images of fractured femurs from the following groups: (**A**) control, (**B**) bFGF, (**C**) Spheroid, (**D**) bFGF-loaded spheroid (bFGF-spheroid), and (**E**) bFGF-loaded dead spheroid (bFGF-dSpheroid). (**F**,**G**) Quantification of callus area and bone mineral content at the fracture site 4 weeks post-fracture. (**F**) Analysis of bone volume (mm^3^) in calluses from the control, spheroid, bFGF, bFGF-loaded spheroid (bFGF-spheroid), and bFGF-loaded dead spheroid (bFGF-dSpheroid) groups. (**G**) Analysis of bone mineral content (mg) in the same groups. Data are presented as mean ± SD (*n* = 5). “a” indicates statistical significance (*p* < 0.05) compared to the control group. “b” indicates statistical significance (*p* < 0.05) compared to the spheroid group.

Figure 4 illustrates the successful injection of the spheroids surrounding the fracture site using a micropipette. Figure 5A–G shows the CT images and the quantified BV and BMC four weeks after a fracture in the high-dose experiments. In the groups treated with bFGF alone (BV, *p* = 0.010; BMC, *p* = 0.023), bFGF-loaded spheroids (BV, *p* = 0.006; BMC, *p* = 0.004), and bFGF-loaded dSpheroids (BV, *p* = 0.032; BMC, *p* = 0.014), both the BV and BMC were significantly higher compared to the control group. There were no significant differences among the bFGF alone, bFGF-loaded spheroids, and bFGF-loaded dSpheroids groups. A high dose of bFGF promoted bone formation regardless of the presence of spheroids.

### 3.4. Effect of Low-Dose bFGF-Loaded Spheroids on Bone Volume and Bone Mineral Content After Fracture

**Figure 6:**
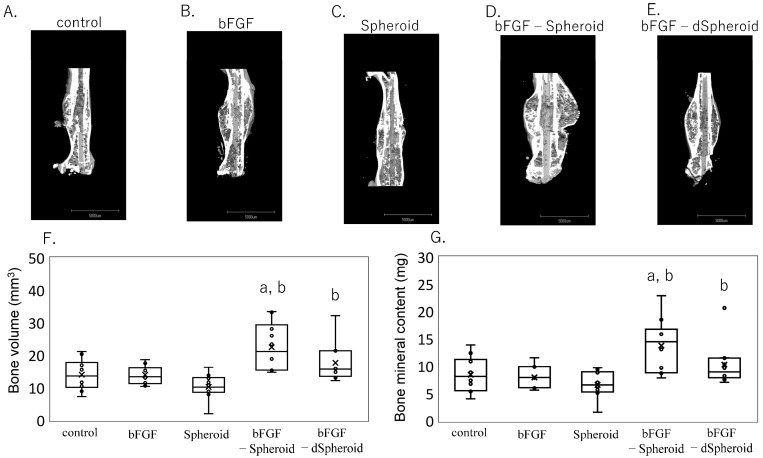
Micro-CT analysis of femurs following transplantation of low-dose bFGF-Loaded spheroids. Representative 3D micro-CT images of fractured femurs from the following groups: (**A**) control, (**B**) bFGF, (**C**) Spheroid, (**D**) bFGF-loaded spheroid (bFGF-spheroid), and (**E**) bFGF-loaded dead spheroid (bFGF-dSpheroid). (**F**,**G**) Quantification of callus area and bone mineral content at the fracture site 4 weeks post-fracture. (**F**) Analysis of bone volume (mm^3^) in calluses from the control, spheroid, bFGF, bFGF-loaded spheroid (bFGF-spheroid), and bFGF-loaded dead spheroid (bFGF-dSpheroid) groups. (**G**) Analysis of bone mineral content (mg) in the same groups. Data are presented as mean ± SD (*n* = 10). “a” indicates statistical significance (*p* < 0.05) compared to the control group. “b” indicates statistical significance (*p* < 0.05) compared to the spheroid group.

Figure 6A–G shows the CT images and the quantified BV and BMC four weeks after a fracture in the low-dose experiments. In the group treated with the spheroids loaded with bFGF, both the BV and BMC were significantly higher compared to the control (BV, *p* = 0.012; BMC, *p* = 0.015), bFGF alone (BV, *p* = 0.012; BMC, *p* = 0.008), and spheroid (BV, *p* < 0.001; BMC, *p* < 0.001) groups. Additionally, in the group treated with the dead spheroids (dSpheroids) loaded with bFGF, both the BV and BMC were significantly increased compared to the spheroid-alone group (BV, *p* = 0.003; BMC, *p* = 0.017). This suggests that even non-viable spheroids loaded with bFGF can promote bone healing to a certain extent, although the effect is more pronounced with viable MSC spheroids loaded with bFGF.

## 4. Discussion

This study aimed to investigate the osteogenic potential of bFGF-loaded MSC spheroids in a mouse fracture model. The results demonstrate that the spheroid culture system significantly upregulated the expression of key trophic factors, such as the bFGF, BMP-2 and VEGF, compared to the monolayer cultures. The binding assay confirmed that the bFGF interact effectively with the MSC spheroids, indicating their potential as carriers for growth factors. In vivo, the spheroids loaded with bFGF significantly increased the BV and BMC of the mice compared to the control group. Importantly, neither a spheroid alone, a bFGF alone, nor the non-viable spheroids loaded with bFGF showed significant improvements, highlighting the essential role of live MSC spheroids in enhancing bone regeneration.

The interaction between the bFGF and collagen carriers has been extensively documented in various studies, which have demonstrated that collagen can serve as an effective delivery vehicle for bFGF [32,37]. Collagen, due to its structural properties and high affinity for the bFGF, allows for a sustained release at the target site, enhancing tissue repair and regeneration. For instance, collagen scaffolds have been successfully used to deliver bFGF in bone healing models, demonstrating enhanced osteogenesis [32,37]. The study’s findings underscore the potential of MSC spheroids as carriers for the bFGF. MSC spheroids produce an ECM rich in collagen [38], which has a high affinity for the bFGF. This binding allows for a more sustained release of bFGF, overcoming one of the main limitations of bFGF therapy—its rapid diffusion and short half-life in vivo. The ability of spheroids to act as a delivery system provides a localized and controlled release of bFGF at the fracture site, which is critical for promoting bone formation over an extended period. Importantly, in our study, the bone healing observed with the non-viable spheroids loaded with bFGF was likely due solely to the presence of a bFGF, as non-viable spheroids do not secrete trophic factors or actively participate in bone regeneration. This suggests that the therapeutic effects in this group were driven by the bFGF rather than any contribution from the non-viable spheroids themselves. In contrast, viable MSC spheroids not only act as carriers but also secrete additional factors like the BMP-2 and VEGF, which are crucial for enhancing bone regeneration. This distinction highlights the critical role of both the bFGF and the bioactivity of live MSC spheroids in promoting effective bone healing.

Our study also showed that the bFGF expression was significantly elevated in the spheroid cultures compared to the monolayer cultures. Previous studies have demonstrated that administering 10 μg of bFGF in a mouse fracture model effectively promotes bone formation, while a 1 μg dose does not induce significant bone regeneration [32]. Consistent with these findings, our results showed that 10 μg of bFGF alone stimulated bone formation, whereas 1 μg of bFGF alone did not enhance bone formation. However, when 1 μg of bFGF was combined with spheroids, there was a significant increase in bone formation. This observation has important implications for bone regeneration strategies, as it suggests that lower doses of the exogenous bFGF may be effective when used in conjunction with spheroid-based delivery systems. Reducing the dosage of bFGF minimizes the risks associated with high-dose treatments, such as thrombocytopenia, renal toxicity, and malignant cell activation [20,21], while still optimizing the regenerative effects of the therapy.

The combination of the bFGF and MSC spheroids exhibited a synergistic effect on bone healing, as evidenced by the significant increases in the BV and BMC in the bFGF-loaded spheroid group. In contrast, the non-viable spheroids loaded with bFGF did not produce a similar effect, indicating that the therapeutic benefits are not solely due to the bFGF but also involve the trophic factors secreted by the viable MSCs. Factors such as the BMP-2 and VEGF, which are upregulated in spheroid cultures, likely play an important role in this enhanced osteogenesis. In addition to the synergistic effects of the BMP-2 and bFGF on bone formation [24,30], studies have also demonstrated the synergistic effects of the bFGF, BMP-2 and VEGF in promoting osteogenesis [39]. This synergistic interaction helps to explain the superior therapeutic outcomes observed in the bFGF-loaded spheroid group compared to the control or non-viable spheroid groups, highlighting the importance of both the viable cells and growth factor interactions for optimal bone regeneration. The bFGF binds to the ECM produced by the spheroid and works in concert with the spheroid-derived factors, amplifying the overall regenerative effect. This synergy between the bFGF and the factors secreted by the MSC spheroids, including their ability to promote angiogenesis and osteogenesis, could provide a more robust and effective approach to bone regeneration compared to traditional carriers. While we have demonstrated the efficacy of bFGF-loaded spheroids, further studies are required to assess the bioavailability and pharmacokinetics of the combination. The exact release kinetics of the bFGF from the spheroids, the local concentration at the fracture site, and the duration of the bFGF’s biological activity remain to be explored. Understanding these parameters is crucial for optimizing therapeutic dosing and maximizing the regenerative potential of the treatment. Studies focusing on how long a bFGF remains bioactive when released from MSC spheroids, its interaction with the extracellular matrix (ECM), and its distribution to target tissues will provide more comprehensive insights into the pharmacokinetics of this combination.

While the results are promising, several limitations must be acknowledged. First, the study primarily focused on short-term outcomes, assessing bone regeneration four weeks post-fracture. Long-term studies are needed to determine whether the effects of the bFGF-loaded spheroids persist and lead to fully functional bone restoration. Second, while we assessed bone regeneration using CT imaging to quantify bone volume and mineral density, we did not include histological examinations in this study. This limits the ability to evaluate the detailed tissue-level changes. Future studies will address this by incorporating a histological analysis to provide a more comprehensive understanding of bone regeneration. Third, this study used a mouse model, and while animal models provide valuable insights, they do not fully replicate the complexities of human bone healing. Further research should include testing in larger animal models and clinical trials to assess the translatability of these findings to human patients. Fourth, while this study demonstrated that spheroids could act as bFGF carriers, it did not explore the optimal dosage or release kinetics of bFGF. Future studies should investigate how to fine-tune the release of a bFGF to maximize therapeutic efficacy while minimizing the potential side effects. Fifth, we did not specifically evaluate the potential side effects during treatment. Our primary focus was on bone regeneration outcomes, but we acknowledge that assessing safety and tolerability is essential. Future research will include a thorough assessment of the side effects to provide a more comprehensive understanding of the treatment’s safety profile. Finally, we did not include gene expression profiling to explore the molecular mechanisms behind bone regeneration. Future studies will aim to address this limitation by analyzing gene expression markers such as Runx2, Sox2, BMP2, Aggrecan, and Col2 at the fracture callus site, which would provide deeper insights into the healing pathways involved.

## 5. Conclusions

In conclusion, this study highlights the potential of bFGF-loaded MSC spheroids as an effective strategy for enhancing bone regeneration. The combination of spheroids and the bFGF showed a clear synergistic effect, significantly increasing bone volume and bone mineral content compared to the controls. The role of viable MSCs in secreting trophic factors such as the BMP-2 and VEGF, alongside the controlled release of the bFGF, suggests that this approach could offer a multifaceted solution to bone repair. While further research is needed to address the limitations of this study, these findings lay the groundwork for developing more effective regenerative therapies for bone healing.

## Figures and Tables

**Table 1:** Primer sequences.

Gene		Sequence (5′-3′)	Product Size (bp)
*bFGF*	F	AGAGCGACCCTCACATCAAG	80
	R	ACGGTTAGCACACACTCCTT	
*Bmp2*	F	AAGGCACCCTTTGTATGTGG	211
	R	GCTAAGCTCAGTGGGGACAC	
*Vegfa*	F	CACTGGACCCTGGCTTTACT	79
	R	TCTGCTCTCCTTCTGTCGTG	
*Gapdh*	F	AACTTTGGCATTGTGGAAGG	223
	R	ACACATTGGGGGTAGGAACA	

## Data Availability

The data supporting the results of this study can be obtained upon request from the corresponding author, K.U.

## Supplementary Materials

The following supporting information can be downloaded at: https://www.mdpi.com/article/10.3390/bioengineering11101041/s1, Figure S1: Analysis of spheroid diameter consistency across three independent experiments..
