# Supplementary material for: Enhancing Bone Formation Through bFGF-Loaded Mesenchymal Stromal Cell Spheroids During Fracture Healing in Mice
Source: Bioengineering (Basel). 2024 Oct 18;11(10):1041. doi: 10.3390/bioengineering11101041 (PMC11504918; doi:10.3390/bioengineering11101041)
Supplement: Supplementary file 1 [file bioengineering-11-01041-s001.zip › bioengineering-3238069-supplementary.pdf]

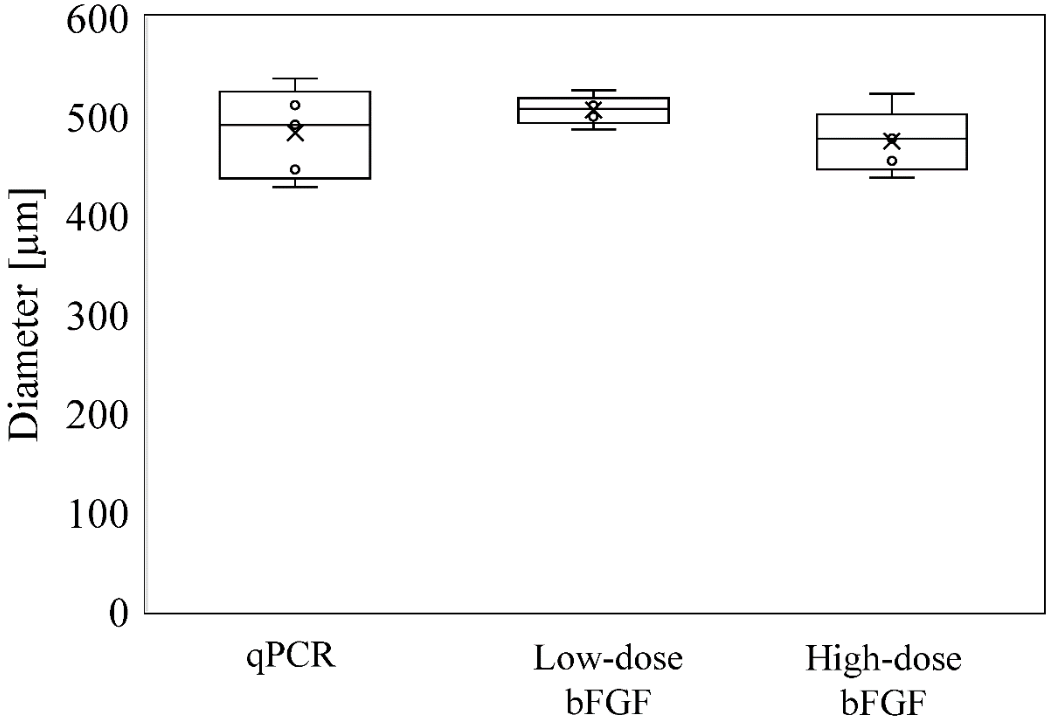

Supplementary Figure S1: Analysis of spheroid diameter consistency across three independent experiments. Five spheroids were randomly sampled from each experiment (for qPCR, low-dose bFGF experiments, and high-dose bFGF experiments). Spheroid diameters were measured using ImageJ after imaging under a light microscope. The results indicate low variability in spheroid formation, with no significant differences in diameter observed among the three groups, demonstrating consistency across experiments.
